# Supplementary material for: Identification of photoperiod-induced specific miRNAs in the adrenal glands of Sunite sheep (Ovis aries)
Source: Front Vet Sci. 2022 Jul 22;9:888207. doi: 10.3389/fvets.2022.888207 (PMC9354845; doi:10.3389/fvets.2022.888207)
Supplement: Supplementary file 1 [file Data_Sheet_1.ZIP › Supplementary file/miRDeep2 file.zip!/miRDeep2 file/Know_Structure/SP42A3/oar-mir-374a.pdf]

miRBase precursor : oar-mir-374a
Total read count : 21625
oar-miR-374a read count : 17659
remaining reads : 3966

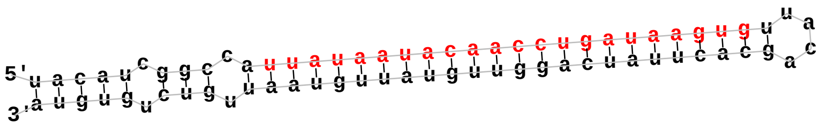

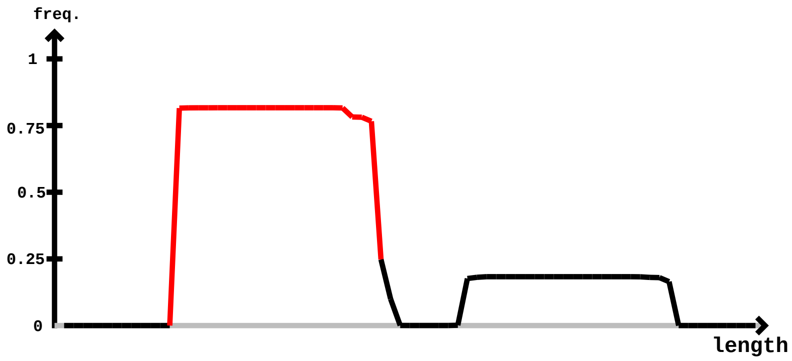

oar-miR-374a

```
5'- uacaucggccauuauaauacaaccugauaaguguuacagcacuuaucagguuguauuguaauugucugugua   -3'    exp
    (((((.(((.((((((((((((((((((((((((....)))))))))))))))))))))))).))).)))))    reads      mm         sample
    ...........uuauaauacaaccugaA............................................    1          1          seq
    ...........Auauaauacaaccugau............................................    1          1          seq
    ...........uuauUauacaaccugau............................................    1          1          seq
    ...........uuauaauacaaccugau............................................    15         0          seq
    ...........uuauaauacaaccugGua...........................................    4          1          seq
    ...........uuauaauacaaccugauU...........................................    11         1          seq
    ...........uuauaauacaUccugaua...........................................    1          1          seq
    ...........uuauUauacaaccugaua...........................................    5          1          seq
    ...........uCauaauacaaccugaua...........................................    1          1          seq
    ...........uuauaauacaaccugaua...........................................    684        0          seq
    ...........uuaCaauacaaccugaua...........................................    8          1          seq
    ...........Cuauaauacaaccugaua...........................................    9          1          seq
    ...........uuaAaauacaaccugaua...........................................    8          1          seq
    ...........uuauaauacaaccugauaa..........................................    21         0          seq
    ...........uGauaauacaaccugauaag.........................................    1          1          seq
    ...........uuauaauacaaccugauaag.........................................    322        0          seq
    ...........uuaCaauacaaccugauaag.........................................    3          1          seq
    ...........uuaCaauacaaccugauaagu........................................    138        1          seq
    ...........uuauaauacaaccugauaagu........................................    11063      0          seq
    ...........uuaCaauacaaccugauaagug.......................................    48         1          seq
    ...........uuauaauacaaccugauaagug.......................................    3148       0          seq
    ...........uuauaauacaaccugauaagugu......................................    2136       0          seq
    ...........uuauaauacaaccugauaaguguu.....................................    4          0          seq
    ............uauaauacaaccugauaagu........................................    6          0          seq
    ............uauaauacaaccugauaagug.......................................    3          0          seq
    ............uauaauacaaccugauaagugu......................................    12         0          seq
    ............uauaauacaaccugauaaguguu.....................................    1          0          seq
    .............auaauacaaccugauaagu........................................    3          0          seq
    .............auaauacaaccugauaagugu......................................    1          0          seq
    ...............aauacaaccugauaaguguuacagc................................    1          0          seq
    ...............aauacaaccugauaaguguuacagcacu.............................    3          0          seq
    ........................................acuuaucagguuguauugu.............    1          0          seq
    ........................................acuuaucagguuguauuAua............    1          1          seq
    ........................................acuuaucagguuguauugua............    1          0          seq
    ........................................acuuaucagguuguauuguaa...........    1          0          seq
```

oar-miR-374a

```
uacaucggccauuauaauacaaccugauaaguguuacagcacuuaucagguuguauuguaauugucugugua
........................................acuuaucagguuguauuguaau..........    10      0      seq
........................................acuuaucagguuguauuguaauu.........    8       0      seq
.........................................cuuaucagguuguauugu.............    9       0      seq
.........................................cuuaucagguuguauugua............    42      0      seq
.........................................cuuCucagguuguauugua............    1       1      seq
.........................................cuuaucagguuguauuAua............    2       1      seq
.........................................cuuaucagguuguauuguaa...........    16      0      seq
.........................................cuuaucagguuguauuguaau..........    310     0      seq
.........................................cuuaucagguuguauuguaauu.........    3408    0      seq
.........................................cuuaucagguuguauuguaauug........    2       0      seq
..........................................uuaucagguuguauuguaau..........    5       0      seq
..........................................uuaucagguuguauuguaauu.........    94      0      seq
..........................................uuaucaAguuguauuguaauu.........    1       1      seq
...........................................uaucagguuguauuguaau..........    3       0      seq
...........................................uaucagguuguauuguaauu.........    47      0      seq
```
